# Effects of periodic photoinhibitory light exposure on physiology and productivity of Arabidopsis plants grown under low light

**DOI:** 10.1093/jxb/erx213

**Published:** 2017-06-29

**Authors:** Yonglan Tian, Joanna Sacharz, Maxwell A Ware, Huayong Zhang, Alexander V Ruban

**Affiliations:** 1 School of Biological and Chemical Sciences, Queen Mary University of London, UK; 2 Research Center for Engineering Ecology and Nonlinear Science, North China Electric Power University, Beijing, China

**Keywords:** Acclimation, Arabidopsis, periodic high light exposure, photoinhibition, photosynthesis, protective non-photochemical quenching

## Abstract

This work examined the long-term effects of periodic high light stress on photosynthesis, morphology, and productivity of low-light-acclimated Arabidopsis plants. Significant photoinhibition of Arabidopsis seedlings grown under low light (100 μmol photons m^−2^ s^−1^) was observed at the beginning of the high light treatment (three times a day for 30 min at 1800 μmol photons m^−2^ s^−1^). However, after 2 weeks of treatment, similar photosynthesis yields (*F*_v_*/F*_m_) to those of control plants were attained. The daily levels of photochemical quenching measured in the dark (*qP*_d_) indicated that the plants recovered from photoinhibition within several hours once transferred back to low light conditions, with complete recovery being achieved overnight. Acclimation to high light stress resulted in the modification of the number, structure, and position of chloroplasts, and an increase in the average chlorophyll *a*/*b* ratio. During ontogenesis, high-light-exposed plants had lower total leaf areas but higher above-ground biomass. This was attributed to the consumption of starch for stem and seed production. Moreover, periodic high light exposure brought forward the reproductive phase and resulted in higher seed yields compared with control plants grown under low light. The responses to periodic high light exposure of mature Arabidopsis plants were similar to those of seedlings but had higher light tolerance.

## Teaser

Arabidopsis plants acclimated to chronic periodic high light stress attained reproductive phase earlier and showed higher productivity than plants grown under low light.

## Introduction

When light levels exceed the capacity of photosynthesis in leaves, the excess absorbed energy can lead to the inactivation of the reaction centers (RCs). Consequently, this can lead to the formation of reactive oxygen species (ROS) and eventually to the reduction of electron transport efficiency and photosynthesis as a whole—the phenomenon known as photoinhibition (Powles, 1984; Osmond and Förster, 2008; Adams *et al.*, 2013). Photoinhibition is manifested by the inefficiency of both photosystem II (PSII) and photosystem I (PSI) quantum yields (Sonoike, 2011; Järvi *et al.*, 2015; Ware *et al.*, 2015). In particular, the inactivation of RCII is due to damage to the D1 protein, a key component of the RC complex. The extent of this damage has been shown to be directly proportional to light intensity (Järvi *et al.*, 2015).

It has been reported that photoinhibition can cause reduced energy and carbon accumulation at the leaf and plant level (Ögren, 1994; Raven, 1994). The photosynthetic activity and sink activity for utilizing photosynthate govern plant productivity (Sun *et al.*, 1999). High light stress not only influences the photosynthetic process but also continuously influences starch synthesis and reproductive growth. This is because starch or carbohydrate mobilization is essential in the control of the floral transition in *Arabidopsis thaliana* (Corbesier *et al.*, 1998). However, previous research produced no evidence that photoinhibited plants possess slower growth rates (Adams *et al.*, 2014). Indeed, seedlings of *Eucalyptus pauciﬂora* and *Eucalyptus nitens* experiencing a high degree of photoinhibition grew better (producing higher biomass and more extensive root growth) than seedlings that displayed less or no photoinhibition (Blennow *et al.*, 1998; Close and Beadle, 2003).

Plants have developed various regulatory mechanisms to acclimate to their light environments (Smith, 1982; Terashima *et al.*, 2005; Adams and Demmig-Adams, 2014; Yamori, 2016). They evolved (i) avoidance techniques at the whole-plant level, including controlled leaf and chloroplast movements (Anderson and Osmond, 1987; Björkman and Demmig-Adams, 1995; Park *et al.*, 1996; Ruban, 2009; Tikkanen *et al.*, 2012); (ii) physiological and molecular alterations of the photosynthetic membrane, such as an increased chlorophyll *a*/*b* ratio, reduced light-harvesting antenna size, increased anthocyanin content, higher content of the cytochrome *b*_*6*_*f* complex, and fewer antenna complexes LHCII and CP24 (Anderson *et al.*, 1988; Chow *et al.*, 1990, 1991; Bailey *et al.*, 2001; Kouřil *et al.*, 2013; Wientjes *et al.*, 2013); and (iii) alteration of leaf anatomy, such as greater stomatal and mesophyll conductance, smaller rosette diameters, thicker leaves, and higher leaf area ratio and leaf mass ratio compared with plants grown under low light (Tyystjärvi and Aro, 1996; Piel *et al.*, 2002; Warren *et al.*, 2007; Yamori, 2016).

The capacity for acclimation from one light environment to another is dependent on the developmental stage of the plant and varies greatly between and within species (Bailey *et al.*, 2001; Borsani *et al.*, 2001; Osmond and Förster, 2008; Carvalho *et al.*, 2015). *Castanospermum australe* showed a decrease in maximal rate of photosynthetic O_2_ evolution (*P*_max_) at higher growth irradiance (Anderson and Osmond, 1987). The shade leaves of *Alocasia macrorrhiza* showed significant capacity for repair and contribution to the whole-plant carbon budget during acclimation to high light (Mulkey and Pearcy, 1992). A study of a number of British plant species suggested that the capacity for photosynthetic acclimation is linked to habitat distribution (Murchie and Horton, 1997). Some plant species have been found to exhibit higher degrees of photoinhibition during juvenile phases (Krause *et al.*, 1995; Carvalho *et al.*, 2015), while other species present more pronounced photoinhibition at the mature stage (Manetas *et al.*, 2002). The effects of high light stress on Arabidopsis seedlings, especially regarding the impact on photosynthesis, have been less well studied. It is still unclear whether Arabidopsis plants have different mechanisms to acclimate to high light stress at different developmental stages.

Nevertheless, the biological significance of photoinhibition should be evaluated in the broader context of long-term photosynthetic production (Ögren, 1994). Prolonged exposure of plants or organisms to excessive radiation may also result in the destruction of the photosynthetic pigments (Powles, 1984; Alves *et al.*, 2002). The longer the duration of high light treatments, the longer are recovery times (Mulkey and Pearcy, 1992). There is a lack of knowledge regarding photoinhibition and acclimation of plants to repeated periodic high light stress, such as large sunflecks or tree-fall gaps (Krause *et al.*, 2001). This is a common occurrence in nature, and therefore acquiring novel knowledge of this would be very important to better understand the *in vivo* ontogenesis of plants.

To the best of our knowledge, there has been no study of the effect of periodic high light stress on photoinhibition and acclimation covering the entire growth period of plants. In this study, low-light-grown wild-type *A. thaliana* plants were exposed to periodic high light at different developmental stages. The objectives of the study were to: (i) investigate the extent of photoinhibition and its recovery following periodic daily high light treatment; (ii) assess the state and tolerance of plants by the photosynthetic yield at different stages of growth; and (iii) understand the effect of long-term periodic high light stress on intracellular chloroplasts, the morphological acclimation of leaves and stems, and plant productivity.

## Materials and methods

### Plant material and growth conditions

The model plant used in this research was wild-type *Arabidopsis thaliana* (Col-0). Before sowing, seeds were sterilized by soaking in 2 ml Eppendorf tubes with 50% ethanol and 0.1% Triton X-100 for 3 min, then washed three times in distilled water and cold treated (4 °C) for 3 days. The soil used was a mixture of 6:6:1 Levington M3 potting compost, John Innes No. 3 soil, and perlite (Scotts UK, Ipswich, UK). The soil was sterilized by autoclaving (15 min at 123 °C).

Plants were grown in Sanyo plant growth cabinets (Panasonic, Japan) for 11 days after sowing, with a 10 h photoperiod at a light intensity of 100 μmol photons m^−2^ s^−1^ and a day/night temperature of 20–24 °C (day)/18 °C (night). It took ~3 days for the seeds to germinate. From the 12th day after sowing, the plants were moved to plant growth shelves with the same photoperiod at a light intensity of 100 μmol photons m^−2^ s^−1^ (provided by T8 fluorescent lamps) and the same day/night temperature. Relative humidity, temperature, and nutrient and water supply were kept constant during the experiment.

### High light treatment

**Fig. 1.**
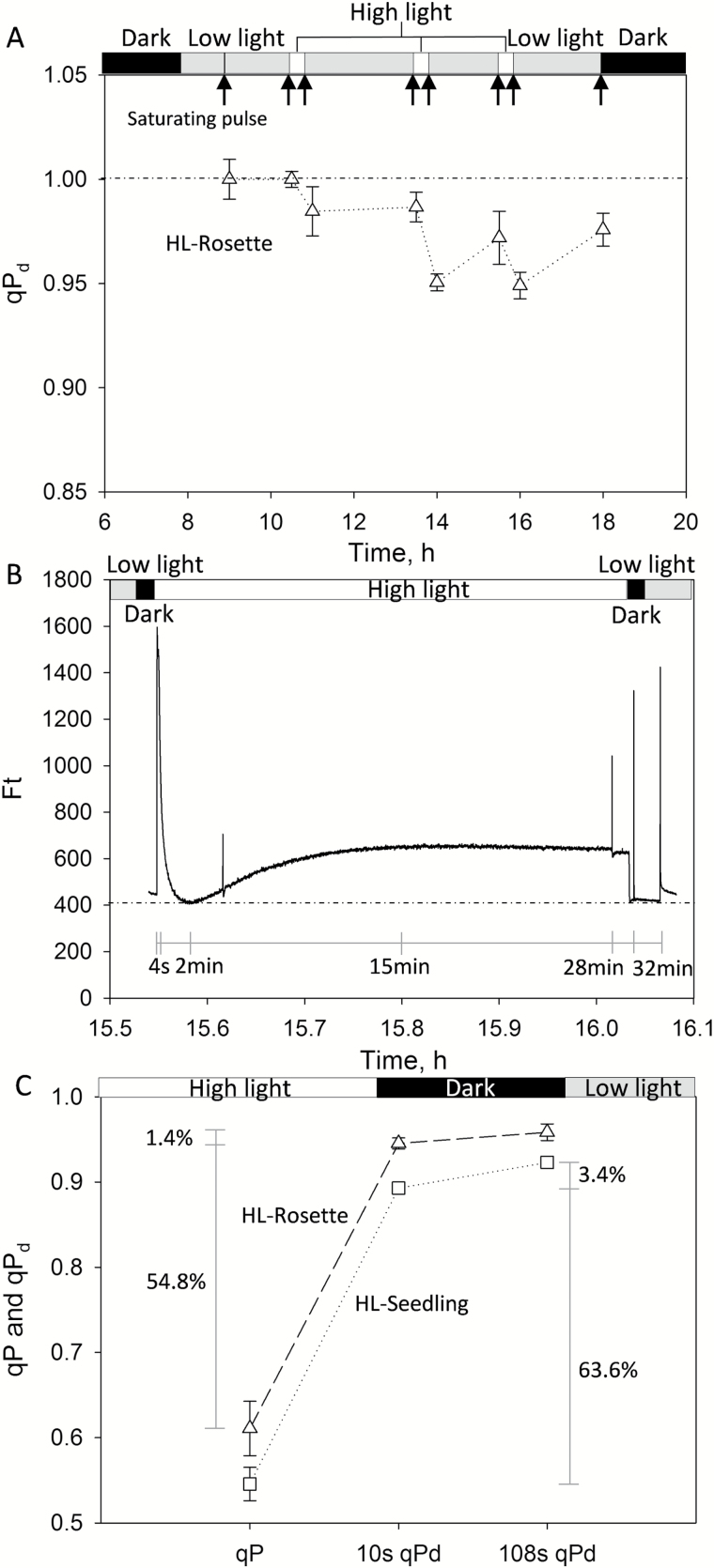
(A) Daily variation of the photochemical quenching measured in the dark (*qP*_d_) parameter of *Arabidopsis thaliana* treated from the rosette stage with high light (HL-Rosette). (B) Changes in chlorophyll fluorescence yield (*F*_t_, relative units) of 6-week-old HL-Rosette plants during and after exposure to high light. Data were collected from the third high light treatment during the day. (C) *qP*_d_ recovery after the third treatment. The photochemical quenching (*qP*) was measured in 6-week-old plants under high light treatment for 28 min (1800 μmol photons m^−2^ s^−1^ illumination); 10 s *qP*_d_ and 108 s *qP*_d_ were the *qP*_d_ measured after 10 s and 108 s in the dark, respectively. Data are presented as mean±SEM (*n*=2).

The growth of *A. thaliana* includes four major stages—germination (seedling), rosette production, bolting (flowering), and senescence (Cotter, 2005). The seedling and rosette stages are vegetative growth stages. During these two stages, the effect of high light stress on photosynthetic process would influence starch accumulation and further impact productivity. Hence, in the present study high light treatments (1800 μmol photons m^−2^ s^−1^) were conducted from the seedling stage (HL-Seedling; the 12th day after sowing) and the rosette stage (HL-Rosette; the sixth week after sowing), respectively. Plants were treated three times per day (at 10.30–11.00, 13.30–14.00, and 15.30–16.00 h, for 30 min at each time). Between the treatments, they were exposed to low light (100 μmol photons m^−2^ s^−1^) in line with the control groups. The period and frequency of the high light treatment were chosen according to a previous study by Mulkey and Pearcy (1992), to give the plants enough time to undergo photoinhibition as well as recovery. The continuous high light was provided by a light device (LightDNA Valoya system; Valoya Oy, Finland), with available spectra varying from 380 to 820 nm. The spectrum of the high light device is shown in Supplementary Fig. 1 at *JXB* online, in which the purple line represents the sum of all light sources together. During the treatment, chlorophyll fluorescence was measured to record the status of the plants, as shown in Fig. 1. The high light intensity was approximately the same as full summer noon sunlight at temperate latitudes (Weston *et al.*, 2000). The photoperiod, day/night temperature, and other growing conditions were the same in the treatment and control groups.

### Chlorophyll fluorescence

Fluorescence measurements were performed on the morning just before the high light treatment to reflect the balance between photoinhibition and repair, integrated over the previous exposure (Pearcy, 1994). All plants were subjected to 45 min dark adaptation before each measurement. Measurements were performed on whole plants with intact leaves. Whole-plant maximum PSII quantum yield (*F*_v_/*F*_m_) was visualized using a variable chlorophyll ﬂuorescence imaging system (Imaging PAM; Walz, Effeltrich, Germany) consisting of a CCD camera, LED lights, and a control unit connected to a PC running dedicated software (ImagingWin 2.3; Walz).

Fluorescence traces were recorded using a Junior-PAM fluorometer (Walz). The leaf clip was applied in order to deliver the light guide to the leaf surface. Standard foil was placed behind the leaf to enable a reproducible optical environment during the measurements. The *qP*_d_ of the high-light-treated plants was measured at the end of the dark phase and before and after each high light treatment during the daytime. Chlorophyll fluorescence induction (two cycles) was recorded for control and treated plants exposed, respectively, to 2 × 5 min 285 or 1150 μmol photons m^−2^ s^−1^ actinic light illumination periods, each followed by 5 min of dark relaxation (see Supplementary Fig. S2) (Belgio *et al.*, 2012). The choice of different actinic light intensities (i.e. 285 and 1150 μmol photons m^−2^ s^−1^) for chlorophyll ﬂuorescence induction in the control and treated plants was made on the basis of the extent of photoinhibition estimated from the new indicator of photodamage, the *qP*_d_ parameter (described below), as measured in the dark after the cycle of illumination (Johnson and Ruban, 2010; Ruban and Murchie, 2012).

Light curves were obtained using the rapid light curve trig-run program (settings: 2, corresponding to the following light intensities: 0, 45, 66, 90, 125, 190, 285, 420, and 625 μmol photons m^−2^ s^−1^, 20 s each) and inbuilt ﬁtting (Platt *et al.*, 1980) of the Junior-PAM. All the measurements were conducted on true leaves rather than cotyledons.

The maximum quantum yield of PSII was defined as *F*_v_/*F*_m_= (*F*_m_*–F*_o_)/*F*_m_. *F*_m_ is the maximum fluorescence in the dark-adapted leaf; *F*_v_=*F*_m_–*F*_o_, where *F*_o_ is the dark fluorescence level before illumination.

Non-photochemical quenching (NPQ) was calculated as (*F*_m_–*F*_m_ʹ)/*F*_m_ʹ, and *qP*_d_ was calculated according to the following equation (Belgio *et al.*, 2012; Ruban and Murchie, 2012; Ware *et al.*, 2014, 2015):

qPd=Fm'−Fo'actFm'−Fo'calc (1)

where *F*_m_ʹ is the maximum fluorescence after actinic light illumination, $F_{\text{o}}{'}_{\text{act}}$ is the measured dark level of fluorescence after illumination, and $F_{\text{o}}{'}_{\text{calc}}$ is the calculated dark fluorescence level according to Oxborough and Baker (1997):

Fo'calc=1(1Fo−1Fm+1Fm') (2)

When excess light damages RCII, the *F*_o_ level rises, thus disguising the true quenching effect of NPQ upon *F*_o_ʹ. At low actinic light intensities, there is normally little discrepancy between $F_{\text{o}}{'}_{\text{act}}$ and $F_{\text{o}}{'}_{\text{calc}}$, indicating that the formula of Oxborough and Baker (1997) works well. However, at higher light exposures, the difference between the two $F_{\text{o}}{'}_{\;}$ values increases. This is due to closure of the damaged RCIIs that causes the increase in $F_{\text{o}}{'}_{\text{act}}$ and is reﬂected in the decline of *qP*_d_ below a value of 1 (Ruban and Murchie, 2012; Ruban and Belgio, 2014; Ware *et al.*, 2015).

### Pigment analysis

The total pigments were estimated by spectrophotometric measurements of an 80% ﬁnal acetone extract of leaves according to Porra’s method (Porra *et al.*, 1989). Absorbance at 646.6, 663.6, and 750 nm was detected to determine the chlorophyll concentrations (mg g^–1^ fresh weight) and chlorophyll *a/b* ratio.

### Confocal microscopy

Confocal microscopy was employed to visualize chlorophyll fluorescence in chloroplasts of both HL-Seedling and HL-Rosette plants after 5 days of treatment. Leaf discs were soaked in 0.9 M aqueous sucrose solution for 2 h in the dark and mounted on glass slides, protected from drying by sealed coverslips. Images were obtained with a Leica TCS-SP5 confocal laser scanning microscope (Leica Microsystems Ltd., Milton Keynes, UK) using a 63× oil immersion objective (NA 1.4). An argon laser at 488 nm was used for chlorophyll excitation and emission spectra were registered in the range of 670–720 nm (wavelength selected by monochromator). Images were recorded at 8-bit resolution (512 × 512 pixels) with laser scanning at 400 Hz and 3× frame averaging. For each sample, 6–10 images were taken with one or two cells in each image, and the number of chloroplasts per cell were counted for both untreated (control) and treated (HL-Seedling and HL-Rosette) plants.

### Productivity measurements

The productivity of the plants was expressed as the weekly above-ground biomass, starch content, and leaf area. The above-ground plants were harvested to measure the fresh above-ground biomass and then dried in an oven at 80 °C for 24 hours to obtain the dry above-ground biomass. The starch contents of the fresh leaves were measured according to a previously reported method (Smith and Zeeman, 2006) at 10.00 h each week before treatment. Photographs of the plants were taken with a ruler as a reference on the day of harvesting plants. The total rosette leaf area (cm^2^) was determined using digital images of the plants, which were processed with Image-Pro Plus 6.0 software (Media Cybernetics, Rockville, MD, USA).

After 12 weeks of growth, the measurements of *F*_v_/*F*_m_, leaf area, biomass, and starch contents were stopped to prevent loss of seeds resulting from frequent transportation. Watering of mature plants was stopped 2–3 weeks before seed collection. After being collected, a sample with ~100 seeds and the total seed weight of one plant (in g) were weighed using a microbalance (Fisher PS-100). Then, the total seed numbers were calculated.

### Statistical analysis

Single-factor analysis of variance (ANOVA) was used to assess the difference between the means of the control and high-light-treated groups. Mean values were separated by the least signiﬁcant difference (LSD) test at 5% and 1% levels of signiﬁcance. The statistical calculations were performed using SigmaPlot12 (Systat Software, Inc., Chicago, IL, USA).

## Results

### Daily *qP*_d_ responses to high light treatments

In the absence of photoinhibition, *qP*_d_=1 and the theoretical and actual yields of PSII are extremely close. However, upon the onset of damaged RCs, *qP*_d_ becomes <1, and the actual and theoretical yields diverge (Ruban and Murchie, 2012; Ware *et al.*, 2014, 2015; Carvalho *et al.*, 2015). Fig. 1 shows the response of HL-Rosette plants to periodic high light treatment. The plants were 6 weeks old and had undergone high light treatment for 1 week. As shown in Fig. 1A, after low light exposure (100 μmol photons m^−2^ s^−1^) for 1 h, the *qP*_d_ of the plants was 1, indicating the absence of photoinhibition. After exposure to high light (1800 μmol photons m^−2^ s^−1^), the *qP*_d_ started to decline below 1, that is, photoinhibition occurred. In low light, the *qP*_d_ of treated plants recovered slightly. This decline–recovery pattern was found for each treatment during a day and for both HL-Seedling and HL-Rosette plants (data not shown). The visible responses of HL-Rosette plants to high light treatment on the first day are presented in Supplementary Fig. S3.

Further increase in the light intensity to ~3000 μmol photons m^−2^ s^−1^ did not change the decline–recovery variation during daily treatment, while treatment with 4000 μmol photons m^−2^ s^−1^ caused chlorosis and dehydration of the leaves, leading to the failure of measurement (data not shown). The lesions on the leaves could have result from the synergistic effect of extreme high light and increased temperature (Pearcy, 1994).

During each 30 min treatment, a sharp increase in chlorophyll fluorescence yield (*F*_t_, relative units) was observed at the onset of high light exposure (Fig. 1B). *F*_t_ then decreased rapidly after only 4 s, reaching the lowest value after about 2 min. The yield then gradually recovered and reached the steady state after about 15 min. The photochemical quenching (*qP*) values were reduced after both 5 min and 28 min high light exposure (data not shown). The reduction of *qP* was accompanied by the increase of NPQ from 0.2 to 1.4 in the first 5 min (data not shown). The NPQ values subsequently decreased to ~0.6 at 28 min, indicating the activation of the photosynthetic apparatus by high light.

HL-Seedling plants were affected more by the high light exposure than HL-Rosette plants. The *qP*_d_ values at the end of the third high light treatment were 0.89 and 0.94 for 6-week-old HL-Seedling and HL-Rosette plants, respectively (Fig. 1C). The *qP*_d_ of the plants increased with time. During a 108 s period, the *qP*_d_ of HL-Seedling and HL-Rosette plants increased by 3.4 % and 1.4%, respectively. These results show that *A. thaliana* was able to repair from photoinhibition after the high light treatment, with full recovery reached after the 14 h night/dark period.

### Photosynthesis yields and rate of electron transport confirm the acclimation of plants to high light

**Fig. 2.**
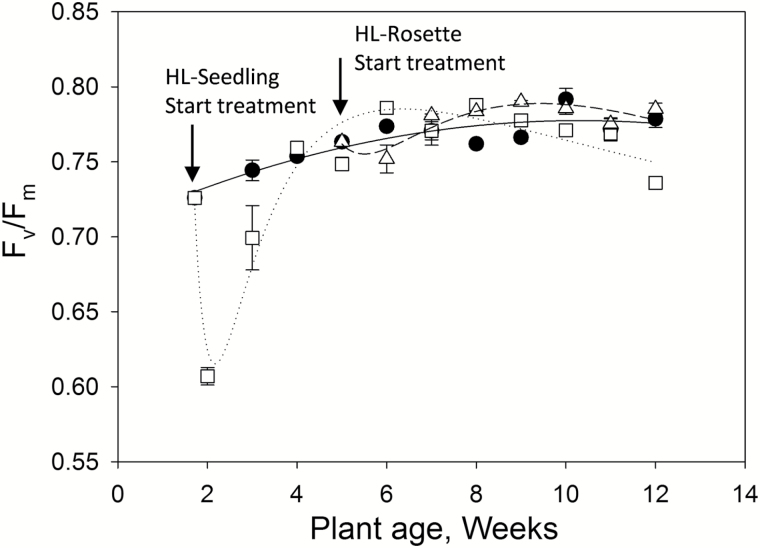
PSII yield (*F*_v_/*F*_m_) of untreated (closed circles) and high-light-treated *Arabidopsis thaliana* plants from the seedling stage (HL-Seedling, open squares) and rosette stage (HL-Rosette, open triangles). Data are presented as mean±SEM (*n*=3).

As shown in Fig. 2, the maximum quantum efficiency of PSII (*F*_v_/*F*_m_) of the untreated plants increased gradually in the first 6–7 weeks, consistent with previously reported data (Carvalho *et al.*, 2015). It then remained stable in the following weeks. The HL-Seedling plants were treated from the 12th day after sowing. The *F*_v_/*F*_m_ values were significantly decreased to 0.60 after 2 days of treatment (*P*<0.01). The *F*_v_/*F*_m_ values increased up to 0.70 after 1 week of acclimation and recovered to the same level as the control plants after 2 weeks of treatment (plant age 4 weeks). HL-Rosette plants were treated from the sixth week after sowing (Fig. 2). After 1 week of treatment, the *F*_v_/*F*_m_ values of HL-Rosette plants (0.75) became significantly lower than the *F*_v_/*F*_m_ values of control plants (0.77; *P*<0.05) and then recovered in the following week (0.78; plant age 7 weeks). The *F*_v_/*F*_m_ variation of HL-Rosette plants followed the same tendency of HL-Seedling plants, but with a smaller amplitude. These results were indicative of the acclimation of plants to the periodic high light exposure, with mature plants acclimating better than seedlings.

**Fig. 3.**
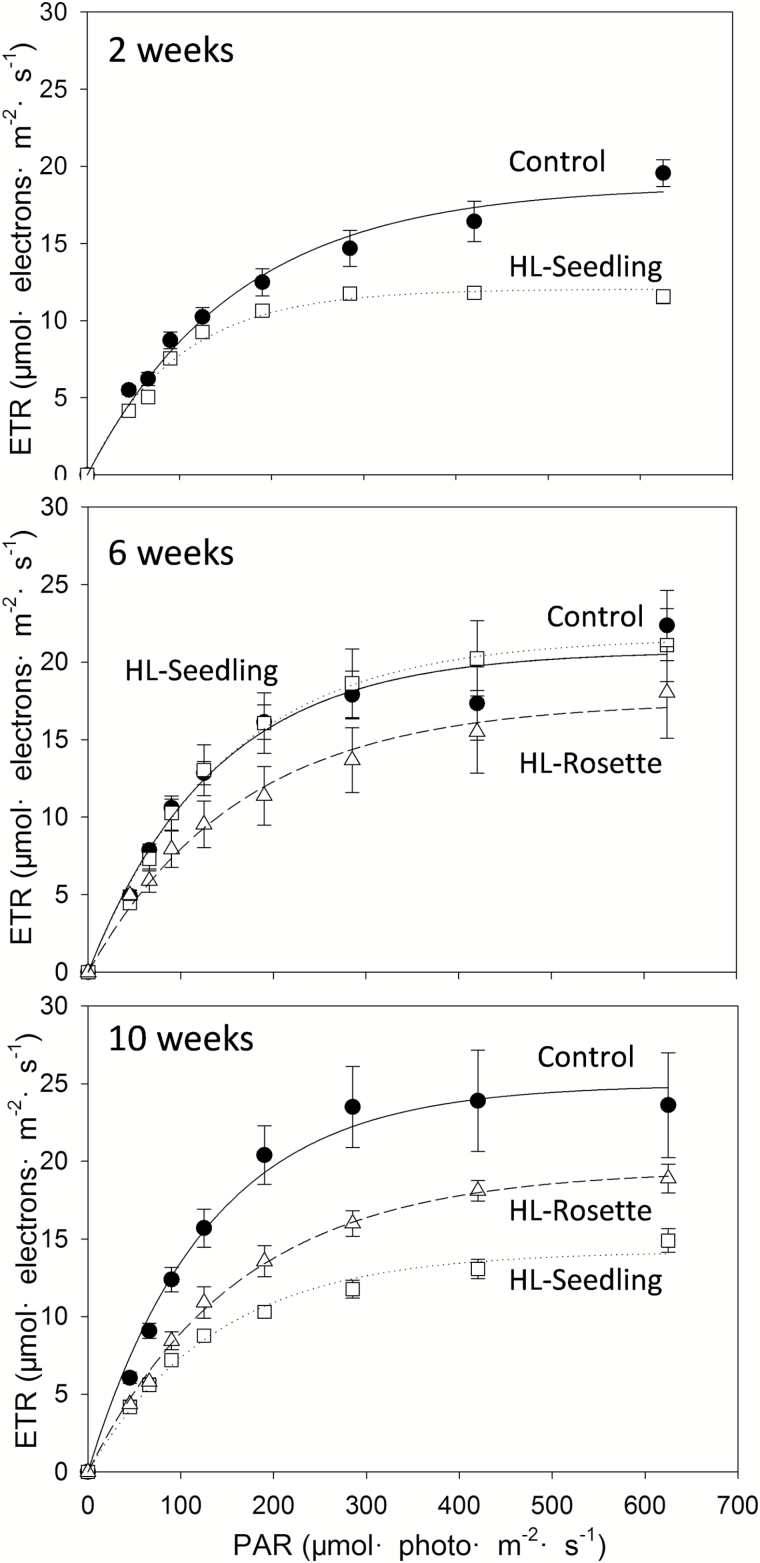
Electron transport rate (ETR) of *Arabidopsis thaliana* treated with high light exposure at plant ages of 2, 6, and 10 weeks. Closed circles, control group; open squares, HL-Seedling; open triangles, HL-Rosette. Lines present regression fit curves [Exponential rise to Maximum, Single, *y*=*a*(1–*e*^−*bx*^) plotted using Sigmaplot 12 (Systat Software, Inc., Chicago, IL, USA)]. Data are presented as mean± SEM (*n*=3). PAR, photosynthetically active radiation.

The acclimation of the electron transport rate (ETR) to high light exposure was also observed (Fig. 3). At the seedling stage (juvenile; plant age 2 weeks), for control plants, the response of the ETR to increasing irradiance followed a saturation-like pattern, increasing under photosynthetically active radiation <625 μmol photo m^−2^ s^−1^ to a maximum of 19.6 µmol electrons m^−2^ s^−1^, while it was only 11.6 µmol electrons m^−2^ s^−1^ for HL-Seedling plants, which were suffering from photoinhibition (Table 1). At the rosette stage (adult; plant age 6 weeks), the HL-Seedling plants were well adapted to the high light treatment and expressed similar ETR responses (21.1 µmol electrons m^−2^ s^−1^) to those of control plants (22.4 µmol electrons m^−2^ s^−1^). However, the HL-Rosette plants, which had been treated for only 2 weeks, showed a lower ETR (18.0 µmol electrons m^−2^ s^−1^) compared with the control plants. This result coincided with the variation of *F*_v_/*F*_m_ values at the same stage. At the reproductive stage (plant age 10 weeks), the leaves of HL-Seedling plants started to wither, while the leaves of control plants were still healthy and ready for reproduction, as shown in Fig. 6. The results for HL-Rosette plants lay between those for the control and HL-Seedling plants. Thus, in line with the results for *F*_v_/*F*_m_ values, the ETR of HL-Seedling plants was lower than that of HL-Rosette plants followed by control plants.

**Table 1.** Electron transport rate (µmol electrons m^−2^ s^−1^) of shade-acclimated and high-light-stressed Arabidopsis thaliana plants at different developmental phases of growth

Group	2 weeks	6 weeks	10 weeks
Control	19.6 ± 0.9	22.4 ± 2.3	23.6 ± 3.4
HL-Seedling	11.6 ± 0.4	21.1 ± 2.4	14.9 ± 0.8
HL-Rosette	–	18.0 ± 2.9	18.9 ± 0.9

Photosynthetically active radiation=625 μmol photo m^−2^ s^−1^. Each value represents the mean±SEM of three measurements from three plants.

### PAM chlorophyll fluorescence quenching analysis

**Fig. 4.**
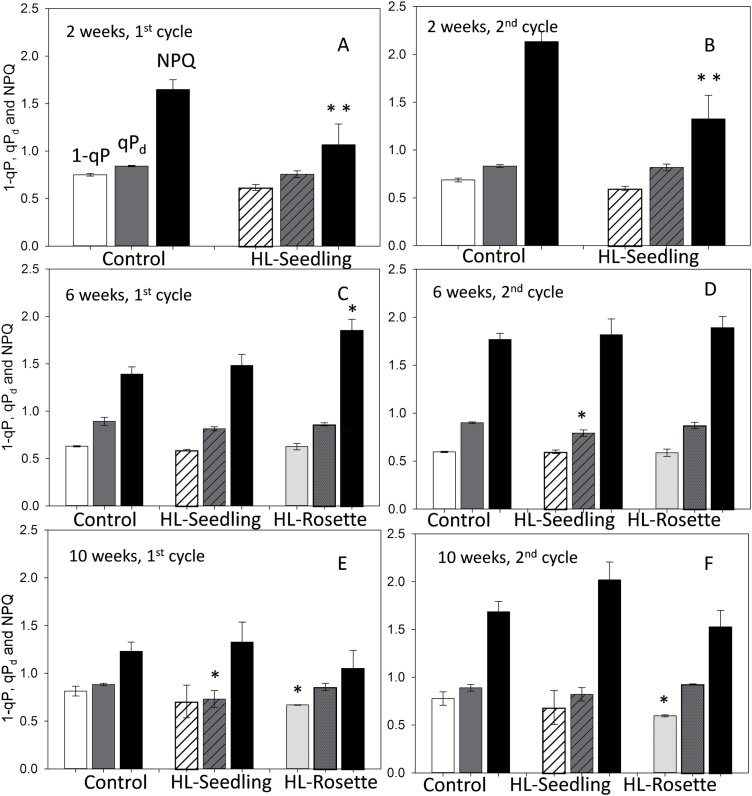
PAM chlorophyll ﬂuorescence analysis of untreated (control) and treated (HL-Seedling, HL-Rosette) *Arabidopsis thaliana* after 2 weeks (A, B), 6 weeks (C, D), and 10 weeks (E, F) of growth, measured with 285 μmol photons m^−2^ s^−1^ actinic light illumination. The excitation pressure (*1*–*qP*, white bars), photochemical quenching measured in the dark (*qP*_d_, dark gray bars), and non-photochemical quenching (NPQ, black bars) after the first and the second illumination cycles are shown. Data are presented as mean±SEM (*n*=3).

Figure 4 presents the calculated parameters from PAM ﬂuorescence induction traces, measured in HL-Seedling, HL-Rosette, and control plants. A typical PAM ﬂuorescence induction trace is shown in Supplementary Fig. S2. The excitation pressure is defined as 1–*qP* and reflects the connected antenna size multiplied by the actinic light intensity. For the high-light-treated plants, this parameter was similar to that in the control group after the first and second illumination cycles during relatively low actinic light illumination (285 μmol photons m^−2^ s^−1^) for almost all growth stages, with HL-Rosette plants being an exception at the 10th week (Fig. 4). When exposed to higher actinic light illumination (1150 μmol photons m^−2^ s^−1^), all tested groups demonstrated higher 1–*qP* values compared with the lower actinic light illumination results (Fig.4; Supplementary Fig. S4). The data in Fig. 4 and Supplementary Fig. S4 were obtained by using the same procedure conducted with different actinic illumination light intensities (i.e. 285 *versus* 1150 μmol photons m^−2^ s^−1^). High-light-treated plants had lower *1*–*qP* values than the control group, especially in HL-Rosette plants (Supplementary Fig. S4). The significantly lower *1*–*qP* of HL-Rosette plants compared with the control plants indicates higher tolerance to subsequent high light stress. Furthermore, high-light-grown plants have smaller antenna systems, which reduces the amount of light being transferred to each RCII. High-light-grown plants also have a better connected antenna system; this means that NPQ is more efficient and protective, which also reduces the amount of excitation pressure on RCII (Ware *et al.*, 2016).

Since the plants were able to recover overnight from photoinhibition, *qP*_d_ did not show significant differences among the different treatments at the age of 6 and 10 weeks at low actinic illumination (285 μmol photons m^−2^ s^−1^). However, when exposed to high actinic illumination, HL-Rosette plants performed better than HL-Seedling plants with regard to light tolerance, showing higher *qP*_d_ values and lower 1–*qP* values.

At the seedling stage (plant age 2 weeks), significantly lower NPQ values of high-light-treated *A. thaliana* were observed at low actinic illumination (285 μmol photons m^−2^ s^−1^) (*P*<0.01; Fig. 4). With the growth of plants (plant age 6 and 10 weeks), the NPQ values of the control group decreased, while the NPQ values of the high-light-treated plants increased (Fig. 4. The NPQ of the HL-Rosette group was significantly higher than that of the control group at the sixth week (*P*<0.05). The trends in NPQ under high and low actinic illumination were similar.

### Variation in chloroplast concentration, number, and structure

**Fig. 5.**
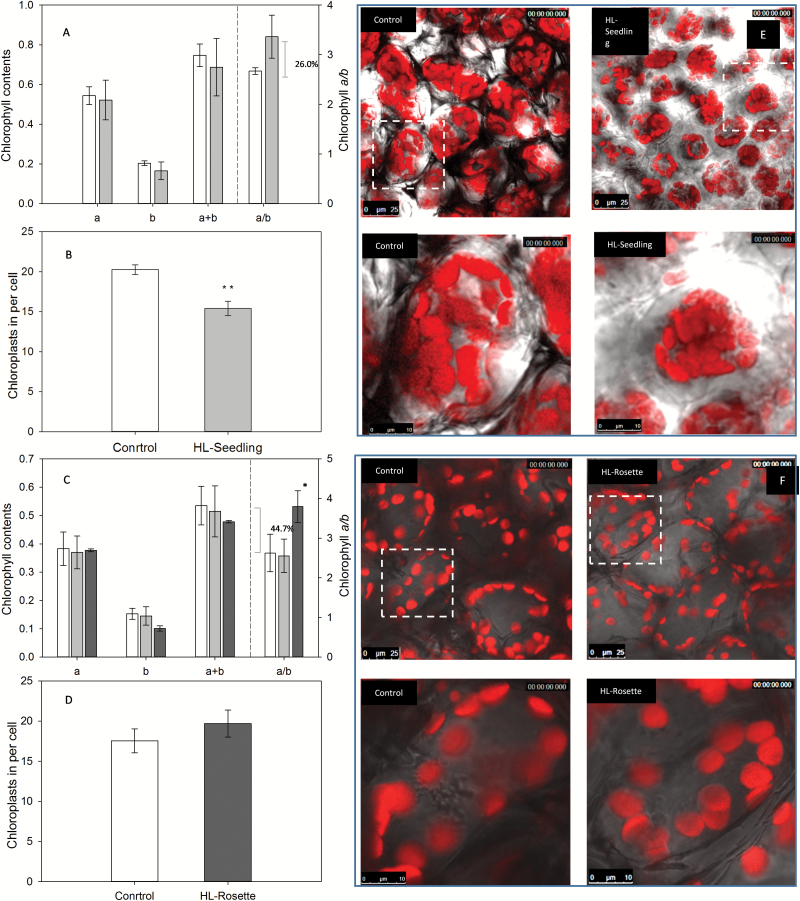
Chlorophyll contents (A, C), chloroplast number (B, D), and confocal chlorophyll fluorescence microscopy (E, F) of untreated (control, white bar), treated from seedling stage (HL-Seedling, light gray bar), and treated from rosette stage (HL-Rosette, dark gray bar) *Arabidopsis thaliana.* (A) Concentrations of chlorophyll *a*, chlorophyll *b*, the chlorophyll *a*/*b* ratio, and total chlorophyll in 17-day-old plants. Data are presented as mean±SEM (*n*=3). (B) Chloroplast number per cell in 17-day-old plants. Data are presented as mean±SEM (*n*=6–10). (C) Concentrations of chlorophyll *a*, chlorophyll *b*, the chlorophyll *a*/*b* ratio, and total chlorophyll in 40-day-old plants. Data are presented as mean±SEM (*n*=3). (D) Chloroplast number per cell in 40-day-old plants. Data are presented as mean±SEM (*n*=6–10). (E, F) Confocal microscopy of intact leaves from (E) 17-day-old and (F) 40-day-old plants, showing fluorescence of chlorophyll in red. Scale bar=25 μm for images of multiple cells and 10 μm for images of a single cell. **P<0.01 between control group and HL-Seedling group according to ANOVA. *P<0.05 between control group and HL-Rosette group according to ANOVA.

The chlorophyll *a*, chlorophyll *b*, and total chlorophyll concentrations were not significantly decreased in either HL-Seedling or HL-Rosette plants (Fig. 5A, C). The chlorophyll *a*/*b* ratio after 5 days of treatment was increased by 26.0% (*P*>0.05) and 44.7% (*P*<0.05) in HL-Seedling and HL-Rosette plants, respectively (Fig. 5A, C). This result is in line with a previous study focusing on chloroplast development under high light (Weston *et al.*, 2000). After 4 weeks of adaption, the chlorophyll content and the chlorophyll *a*/*b* ratio were similar in HL-Seedling plants and control plants (Fig. 5C). Krause *et al.* (2006) found similar results in *Calophyllum longifolium* after acclimation to high light irradiance.

The number of chloroplasts per cell of HL-Seedling plants after 5 days of treatment was significantly decreased compared with control plants (Fig. 5B, *P*<0.01). The study also revealed remarkable alterations in the location of chloroplasts in the HL-Seedling plants compared with control plants (Fig. 5E). Chloroplasts seemed to form dense clusters in the center of the cells in the HL-Seedling group, whereas they were more peripherally located in the control plants (Fig. 5E). However, the number and structure of chloroplasts were not significantly different between HL-Rosette and control plants (Fig. 5D, F). A further increase in the intensity of high light treatment to 4000 μmol photons m^−2^ s^−1^ resulted in a significantly decreased number of chloroplasts in both HL-Seedling and HL-Rosette plants compared with control plants after 5 days of periodic treatment (see Supplementary Fig. S5A). Interestingly, distinct changes in chloroplast structure, within the grana, were visible in the sparsely distributed chloroplasts in HL-Seedling plants (shown as particles) treated with high light at 4000 μmol photons m^−2^ s^−1^ (Supplementary Fig. S5B). Additionally, chloroplasts in HL-Rosette plants were packed more densely when exposed to 4000 μmol photons m^−2^ s^−1^ compared with HL-Rosette plants treated with 2000 μmol photons m^−2^ s^−1^ (Supplementary Fig. S5C).

### High light treatment increased biomass and induced earlier maturation

**Fig. 6.**
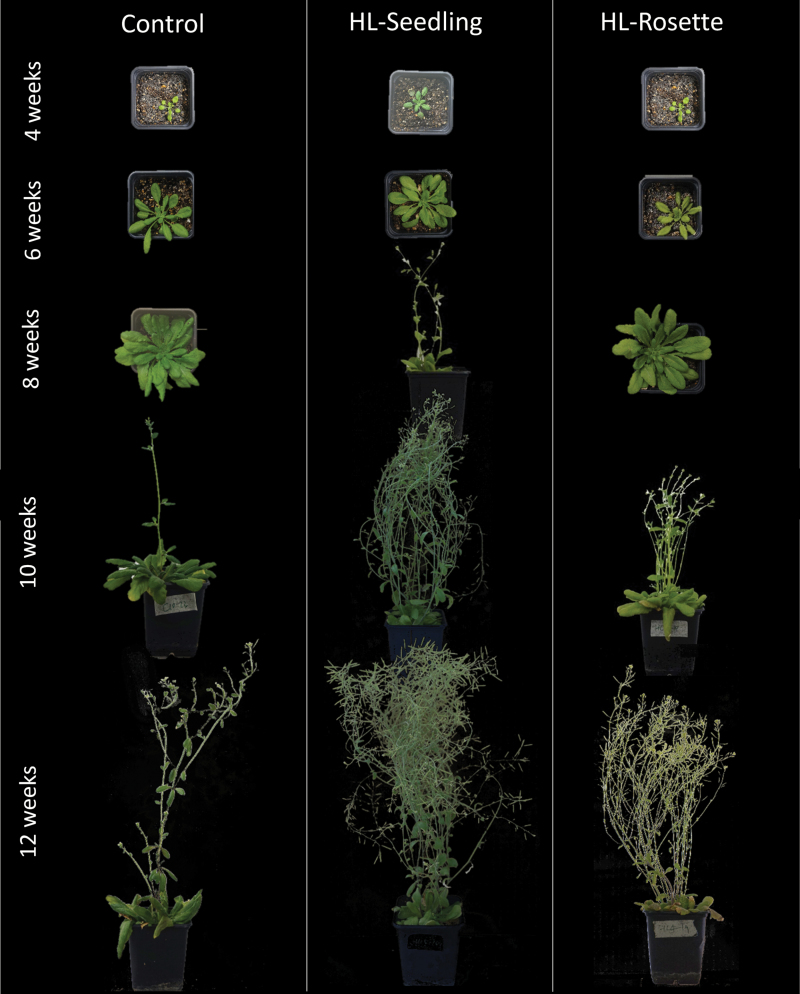
Appearance of *Arabidopsis thaliana* plants treated with high light, at 4, 6, 8, 10, and 12 weeks of growth.

Digital images of whole *A. thaliana* plants are shown in Fig. 6. Although the PSII quantum yields did not change significantly between the fourth and 12th week, the overall impact of high light on plant growth were apparent. HL-Seedling plants started flowering from week 5–6 and produced seeds from week 9. The control plants and HL-Rosette plants both started flowering from week 8–9 and seed production from week 11. Furthermore, high-light-treated plants in both the HL-Seedling and HL-Rosette groups grew more stems than control plants. At week 12, the average number of stems per plant was 2 for the control group, 10 for HL-Seedling, and 12 for HL-Rosette.

**Fig. 7.**
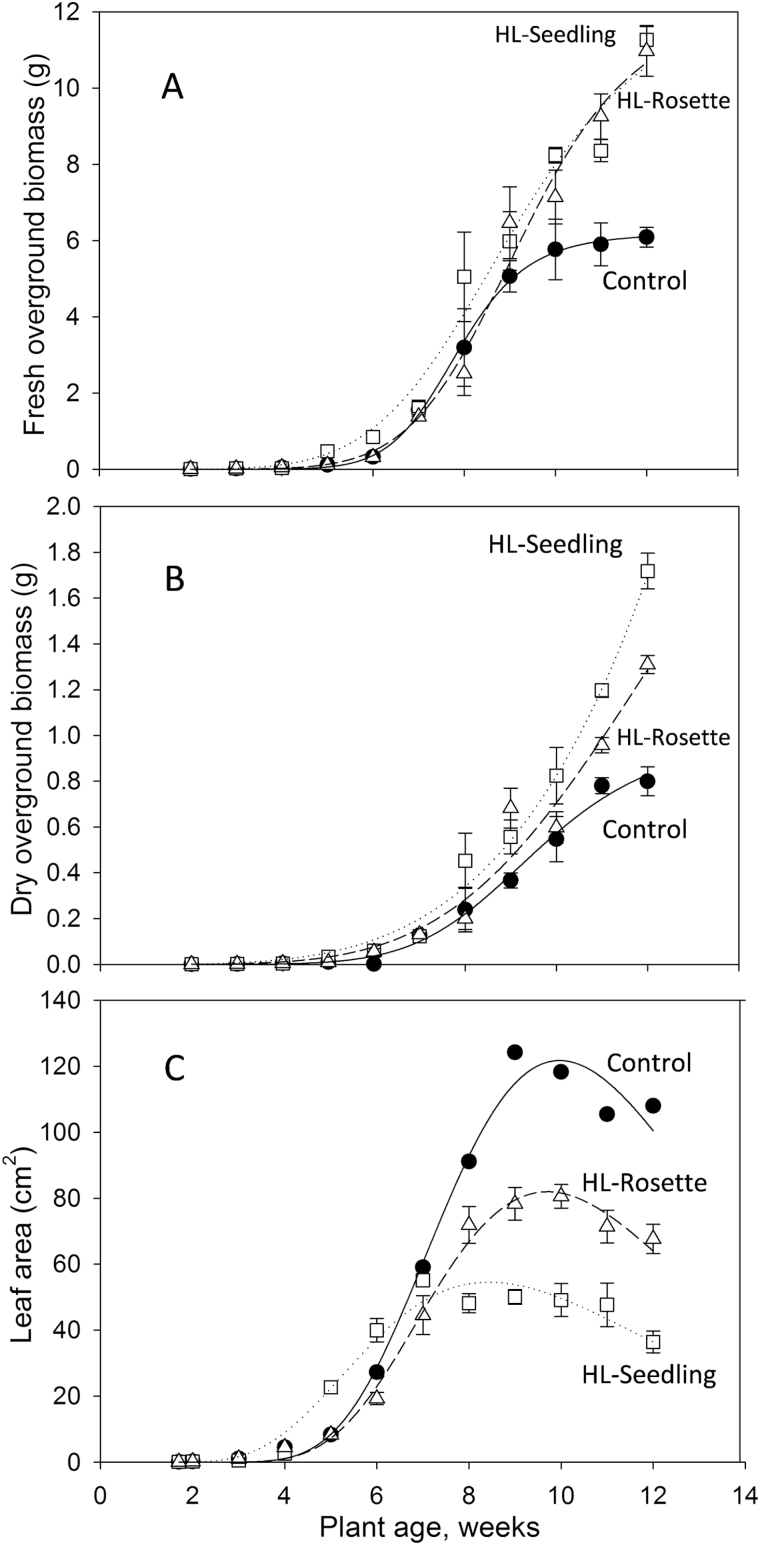
The fresh above-ground biomass (A), dry above-ground biomass (B), and leaf area (C) of *Arabidopsis thaliana* were increased by high light treatment at the seedling stage (HL-Seedling, open squares) and rosette stage (HL-Rosette, open triangles). Closed circles indicate the control group. Data are presented as mean±SEM (*n*=3). Lines present regression fit curves (sigmoidal, Sigmoid, 3 Parameter *y*=*a*/[1+exp(*–*(*x–x*_*0*_)/*b*)]) plotted using Sigmaplot 12 (Systat Software, Inc., Chicago, IL, USA).

The fresh and dry above-ground biomass of the plants increased during the growth period (Fig. 7). The increase was initially slow and became evident after 6 weeks of growth. This tendency was consistent with a previous study on wild-type *A. thaliana* (Caspar *et al.*, 1991). Both fresh and dry above-ground biomass were increased as a result of high light treatment, as shown in Fig. 7. The fresh above-ground biomass of both the HL-Seedling and HL-Rosette groups was similar at the end of the experiments, and was in both cases significantly higher than that of the control group (*P*<0.01) (Fig. 7A). The dry above-ground biomass of HL-Seedling plants was significantly higher than that of HL-Rosette plants (*P*<0.01), which was higher than that of the control group (*P*<0.01) (Fig. 7B).

In spite of promoting biomass, the impact of high light on the leaf area (Fig. 7C) was more associated with the ontogenetic stages (Fig. 6). During the first 6 weeks, HL-Seedling plants showed a higher leaf area than the control group. During later stages, however, high light exposure was associated with a decrease in the leaf area of HL-Seedling and HL-Rosette plants relative to control plants (Fig. 7C). The leaf area of the control group was significantly higher than that of the HL-Rosette plants (*P*<0.01), and the leaf area of HL-Rosette was higher than that of HL-Seedling plants (*P*<0.05). Furthermore, all three tested groups expressed a similar “peak” tendency during the entire growth period, that is, an initial increase in total leaf area and a subsequent decrease. The peak appeared earlier in HL-Seedling plants compared with the control and HL-Rosette groups, in line with an earlier start of the reproductive phase, as indicated in Fig. 6.

### High light treatment markedly decreased starch contents in rosette leaves and enhanced seed production

**Fig. 8.**
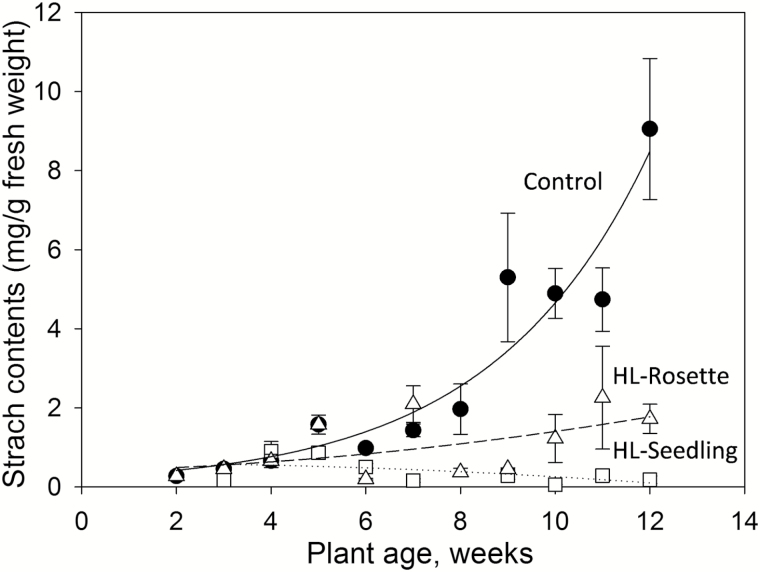
Starch contents of untreated (control group, closed circles) and high-light-treated *Arabidopsis thaliana* leaves from the seedling stage (HL-Seedling, open squares) and rosette stage (HL-Rosette, open triangles). Data are presented as mean±SEM (*n*=3).

It is known that in wild-type plants, leaves of all ages contain a similar amount of starch at the end of the day and starch granules increase significantly in size when plants are kept in continuous light for long periods (Zeeman *et al.*, 2002). Thus, we measured the starch contents of the well-developed outer leaves, which were also used for chlorophyll fluorescence measurement at the same time for each sampling. Unlike the leaf area and biomass, which increased with the growth of plants, the starch content in high-light-treated *A. thaliana* was low during the entire growth period (Fig. 8). During the first 8 weeks, the plants in all three groups had similar starch contents before reaching the reproductive stage, at <2.0 mg g^–1^ fresh weight. This value was consistent with a previous report on starch content in wild-type *A. thaliana* (Caspar *et al.*, 1991). After the eighth week, when the reproductive stage started, the starch content in the control group increased rapidly above 2.0 mg g^–1^ fresh weight (*P*<0.05), reaching a peak of 9.1 mg g^–1^ fresh biomass at the 12th week.

**Fig. 9.**
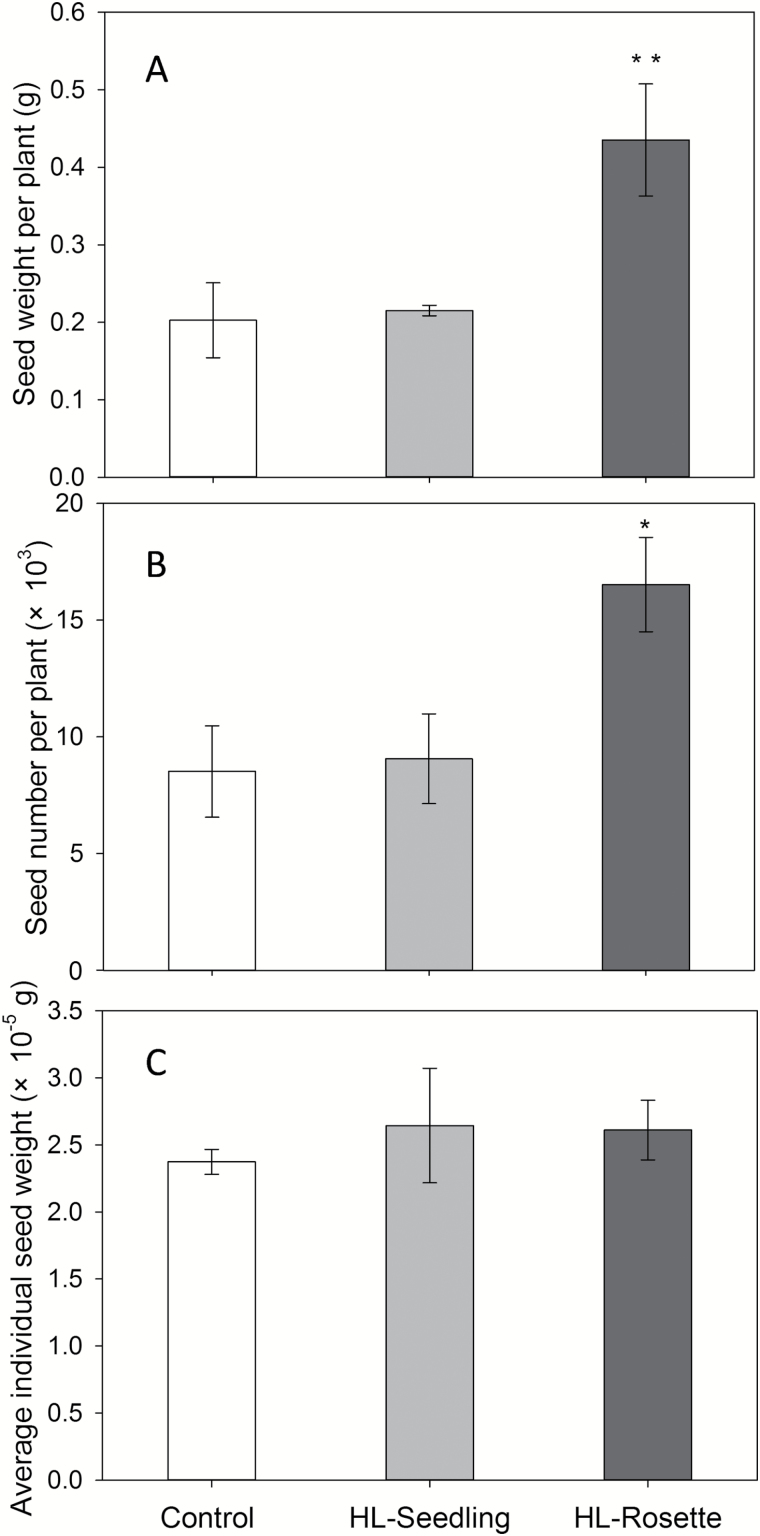
Total seed weight (A), total seed number (B), and average individual seed weight (C) of *Arabidopsis thaliana* plants exposed to high light at the seedling (HL-Seedling) and rosette (HL-Rosette) stage. Data are presented as mean±SEM (*n*=3, 4). Significant differences between control and treated groups according to ANOVA: *P<0.05, **P<0.01.

In accord with the increase in the number of stems (Fig. 6), there was also an increase in productivity in high-light-treated plants, which was reflected by both seed weight and seed number (Fig. 9A, B). Moreover, the individual seed weight was similar in high-light-treated plants and the control group (Fig. 9C). Nevertheless, the difference between HL-Seedling plants and the control group was not significant, although the difference in above-ground biomass was (Fig. 7C). The total seed weight (significance *P*<0.01) and number (significance *P*<0.05) of HL-Rosette plants were almost double the respective values of the control plants. Comparisons of the results of photosynthetic yields and starch contents indicated that *A. thaliana* was able to maintain a similar level of photosynthesis under periodic high light treatment without apparent photoinhibition. However, more starch was consumed for reproduction rather than vegetative growth.

## Discussion

*qP*_d_, reflecting the closure of RCs in the dark, is used in large-scale measurements as a convenient, quickly acquirable parameter that tracks photoinhibition (Ruban and Belgio, 2014; Ware *et al.*, 2015). The decline of *qP*_d_ due to high light treatment indicates the onset of photoinhibition. The rapid increase and decrease of chlorophyll fluorescence yield (*F*_t_) may be associated with the transient increase and decrease in assimilation, reflecting an increase in the ribulose-1,5-bisphosphate concentration available to Rubisco (Pearcy, 1994). After moving back to the low light environment, *qP*_d_ was able to gradually recover (Fig. 1C). The *qP*_d_ was then recovered completely by the next morning; hence, there is an efficient RCII restoration process in operation, which ensures that full photosynthetic efficiency is regained no later than the next morning in the absence of light stress. This finding was in line with an earlier study by Ögren (1994).

Photoinhibition is frequently manifested as a long-term decrease in potential quantum efficiency of PSII (*F*_v_/*F*_m_) (Sobrado, 2011) and occurred at the beginning of high light treatment in the present study, reflected by the decrease in *qP*_d_ and *F*_v_/*F*_m_ (Figs 1 and 2). The reductive effects on *F*_v_/*F*_m_ were registered in different species exposed to excess high light irradiance (Krause *et al.*, 2006, 2012; Sobrado, 2011). Lower ETRs, combined with lower slopes of the linear portion of the light response curve of photosynthesis (Fig. 3), manifested as the inactivation and/or disassembly of PSII cores, especially the D1 protein (Adams *et al.*, 2008). Along with the acclimation to high light exposure, the *F*_v_/*F*_m_ of the plants was able to recover (Fig. 2), which can be attributed to either the increased resistance to photoinhibition or the enhanced capacity for repair (measured as synthesis of D1 protein) (Pearcy, 1994).

Young mutant plants lacking chlorophyll *b* have been reported to possess lower levels of NPQ (Jahns and Krause, 1994). This might explain the lower NPQ of the HL-Seedling group at the second week measured by PAM chlorophyll fluorescence (Fig. 4). At the age of 6 weeks, the chlorophyll contents of HL-Seedling and control group plants reached similar levels which is in line with previous results (Krause *et al.*, 2006). This can be attributed to acclimation processes. The acclimation of photosynthetic pigments to high light has also been determined in trees (Krause *et al.*, 2012). At the 10th week, when the rosette leaves of plants entered the phase of senescence, the NPQ decreased. This result was in agreement with previous research, in which the photoprotective component of NPQ (pNPQ) and ETR were age-dependent, with the maximum pNPQ values observed in the reproductive phase and with lower values during juvenile and senescent phases (Carvalho *et al.*, 2015). However, the NPQ values of the HL-Rosette group were not decreased in spite of the reduction of chlorophyll *b* contents, as shown in Fig. 5C. This correlated with the response of chloroplasts in HL-Rosette plants to high light stress. The number and position of chloroplasts were similar in HL-Rosette and control plants, whereas there was a significant response to high light in the HL-Seedling group: the number of chloroplasts was significantly decreased and they were arranged in dense clusters in the center of the cells to avoid absorbing excess light energy (Fig. 5B, E). The intracellular position of chloroplasts was suggested to be involved in balancing light utilization to counteract high light stress. When exposed to intense light, chloroplasts can rearrange so that they can self-shade each other along the lateral (anticlinal) cell surfaces to lessen the absorption of excess light (Logan *et al.*, 2014).

There have been many claims that photoinhibition is likely to lead to a decrease in carbon gain (CO_2_ assimilation) and reduced plant productivity. However, in reality, long-term downregulation of PSII may be a means by which plants sustain photoprotection (Adams *et al.*, 2008). Moreover, the light intensities that can be tolerated by leaves are strongly dependent on the plant age and leaf developmental stage (Carvalho *et al.*, 2015). *A. thaliana* at the rosette stage acclimated more quickly than plants at the seedling stage, as indicated by the results for *F*_v_/*F*_m_ (Fig. 2). Similar results have previously been reported in *Laminaria saccharina* Lamour (Hanelt *et al.*, 1997).

The treated plants, which were exposed regularly to high light, had a lower total rosette leaf area but higher biomass compared with the control plants (Fig. 7). The decrease in leaf area correlated with the increase in leaf mass per area, as also reported in previous studies (Thompson *et al.*, 1992; Krause *et al.*, 2012). When the plants suffered photoinhibition induced by high light, their starch contents were decreased and they showed low photosynthetic efficiency; this was observed at week 2–3 for the HL-Seedling group and week 6 for the HL-Rosette group (Fig. 8). After acclimation to high light, the starch contents increased at week 4–5 for the HL-Seedling group and week 7 for the HL-Rosette group (Fig. 8), along with increased leaf biomass (Fig. 7), as the high photosynthetic efficiency was likely to enhance the leaf starch content (Mitchell *et al.*, 1997; Sun *et al.*, 1999; De Groot *et al.*, 2001).

However, the accumulation of starch and carbohydrates in the leaves would lead to concomitant feedback down-regulations, including (i) photosynthetic CO_2_ ﬁxation, (ii) thylakoid proteins (the oxygen-evolving complex and D1) responsible for electron donation to and transport in PSII, and (iii) chlorophyll-binding proteins and chlorophyll (in some, but not all, plant species), as well as up-regulation of photoprotective thermal energy dissipation (Adams *et al.*, 2013). The levels of D1 protein decreased dramatically under low light when spinach leaves were supplemented with glucose (Kilb *et al.*, 1996). Therefore, the requirement of starch transfer, that is, carbon export, arose to prevent the disadvantage of feedback down-regulation. The carbon export capacity was typically higher in leaves acclimated to high light than in transferred shade leaves (Adams *et al.*, 2008). In our study, this is reflected by the premature onset of the reproductive stage at week 5–6 for the HL-Seedling group and week 8–9 for the HL-Rosette group (Fig. 6). Earlier maturation was also reported in wheat grown under full light grown in comparison to low-light-grown plants (Mengel and Judel, 2006).

Both control and high-light-treated plants accumulated a certain range of starch content before the onset of the reproductive stage. In detail, the starch content in the leaves of the HL-Rosette and control plants was ~2 mg/g fresh weight at week 7 and 8, respectively. These two groups then started to grow stems (i.e. the beginning of the flowering stage) in the following week, that is, at week 8 and 9 for the HL-Rosette and control group, respectively. The flowering stage of HL-Seedling plants began from week 6, with higher starch contents being observed in week 5. Compared to a previous study, it seems that starch or carbohydrate mobilization was essential in the control of the floral transition in *A. thaliana* (Corbesier *et al.*, 1998), rather than maintenance of a large and available pool of starch in the leaves. Moreover, the level of starch storage in the leaves for activating the floral transition was ~2 mg/g fresh weight; this needs to be further investigated with regard to different growing environments.

Starting from the reproductive phase, due to the export of carbon from rosette leaves, a decrease of starch contents in rosette leaves and an apparent increased number of stems was observed in high-light-treated *A. thaliana* plants (Fig. 8). The stem number and above-ground biomass of the plants was in general highest in HL-Seedling plants, with lower levels in HL-Rosette, and lower still in control group plants (Fig. 6, Fig. 7A, B). In contrast to the control plants, the high-light-treated plants were likely to consume more photosynthetic products for reproductive growth rather than vegetative growth.

In line with the increased number of stems, the seed productivity of the high-light-treated plants was higher than that of the control plants, with significant increases in the seed number and total seed weight being observed for the HL-Rosette group (Fig. 9A, B). This result was in agreement with a previous study on wheat, in which low light intensity decreased the grain and straw yield (Mengel and Judel, 2006). Comparing the total above-ground biomass and seed weights, it seems that the HL-Seedling plants utilized the excessive light energy for stem growth, while the HL-Rosette plants used this energy for seed production.

Acclimation to the photoinhibitory light exposure resulted in changes in starch redistribution and storage. The mobilization of starch in the high-light-treated plants led to earlier maturation, faster growth, a greater number of flowering stems, higher above-ground biomass, and higher seed production in comparison to the control plants. The decrease of starch contents in the rosette leaves was probably associated with the conversion of triose phosphate (triose-P) (Sun *et al.*, 1999; Walters *et al.*, 2004). Triose-P may be converted preferentially into sucrose at lower rates of triose-P production, with increased partitioning to starch synthesis occurring as sucrose synthesis reaches saturation (Sun *et al.*, 1999). Sucrose was dramatically reduced in the light by being exported to non-leaf tissues or reintroduced into the metabolism (Walters *et al.*, 2004). The newly synthesized starch then broke down and provided the substrates needed for sucrose export to the rest of the plant for growth (Walters *et al.*, 2004).

Arabidopsis plants grown under a continuous light intensity of 650 μmol photons m^−2^ s^−1^ showed a 2-fold increase in the seed yield, a 40% increase in mass per seed, and a 60% increase in oil per seed compared with plants grown at 100 μmol photons m^−2^ s^−1^ illumination (Li *et al.*, 2006). In contrast, the mass per seed was not increased by regular high light treatment in the present study (Fig. 9C). According to our observations, when wild-type *A. thaliana* was grown under continuous 400 μmol photons m^−2^ s^−1^ illumination (data not shown), the pods of the seeds were slightly thicker than the pods of plants grown at 100 μmol photons m^−2^ s^−1^ illumination. Both the leaves and the pods of the plants under 400 μmol photons m^−2^ s^−1^ illumination became purple. The different performances of high-light-treated and control leaves and pods may or may not be due to the increase in synthesis of anthocyanins (Szymańska *et al.*, 2014) and the absorption of blue light. In this experiment, the anthocyanin contents were not measured as there was no visible change in leaf color or pod thickness in regular high light treated plants or control plants (grown at 100 μmol photons m^−2^ s^−1^). The impacts of accumulation of anthocyanins and the absorption of blue light need to be taken into account in future research.

Therefore, combining all the morphological, physiological, and photosynthetic parameters, *A. thaliana* was able to acclimate to the periodic high light treatment and increased in productivity. The observations presented here will be beneficial for regulating optimal light conditions for different crop plants. For example, vegetables with edible leaves should be grown under low light for vegetative growth, while plants grown for their fruits could benefit from being grown under low light with periodic exposure to high light intensities in order to increase the yield of fruits/seeds.

## Conclusions

High light exposure damaged PSII. In spite of photoinhibition, Arabidopsis plants were able to overcome the stress of high light and recovered (although not completely at the beginning) once being moved to the dark or low light irradiance. The periods of acclimation were ~2 weeks for seedling plants and <1 week for rosette plants. Acclimation to the photoinhibitory light exposure resulted in changes in starch redistribution and storage, earlier maturation, and higher seed production in comparison to the control plants. The mobilization of starch led to limitation of the growth of rosette leaves and the fast growth and large numbers of flowering stems, resulting in higher above-ground biomass in comparison with controls. Mature Arabidopsis plants performed better in tolerating the high light stress and achieved the highest seed yield, followed by Arabidopsis plants treated from the seedling stage, which in turn had a higher yield than the low-light-grown plants. The experiments showed that periodic photoinhibitory light stress triggers a systemic response in plants that leads to better physiological performance, faster growth, higher biomass, and greater seed production.

## Supplementary data

Supplementary data are available at *JXB* online.

Fig. S1. The spectrum of the high light device.

Fig. S2. Typical PAM ﬂuorescence induction traces of *Arabidopsis thaliana* exposed to two actinic light illumination periods, each followed by dark relaxation.

Fig. S3. Visual responses of *Arabidopsis thaliana* plants on the first day of treatment by high light.

Fig. S4. PAM chlorophyll ﬂuorescence analysis of untreated and treated *Arabidopsis thaliana* leaves after 2, 6, and 10 weeks’ growth.

Fig. S5. Chloroplast number and microscopic appearance of high-light-treated *Arabidopsis thaliana* leaves from seedling and rosette stage.

## Supplementary Material

Supplementary_Figures_S1_S5
