# Supplementary material for: Effects of periodic photoinhibitory light exposure on physiology and productivity of Arabidopsis plants grown under low light
Source: J Exp Bot. 2017 Jun 29;68(15):4249–62. doi: 10.1093/jxb/erx213 (PMC5853873; doi:10.1093/jxb/erx213)
Supplement: Supplementary_Figures_S1_S5 [file erx213_suppl_supplementary_figures_s1_s5.pdf]

Supplementary Materials

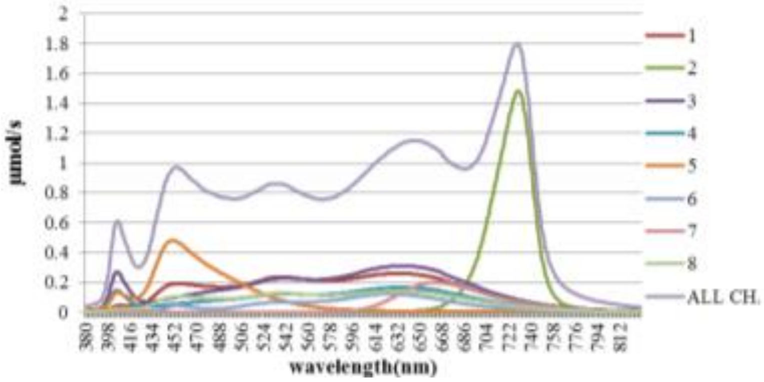

Figure S1. The spectrum of the high light device (LightDNA Valoya system, Valoya Oy, Finland) with the purple line as the sum of all light channels together.

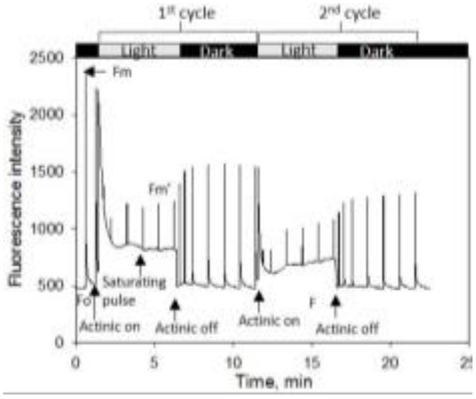

Figure S2. The typical PAM fluorescence induction traces of *Arabidopsis thaliana* exposed to 2×5 min 285 µmol photons m^{-2} s^{-1} actinic light illumination periods, each followed by 5 min period of dark relaxation.

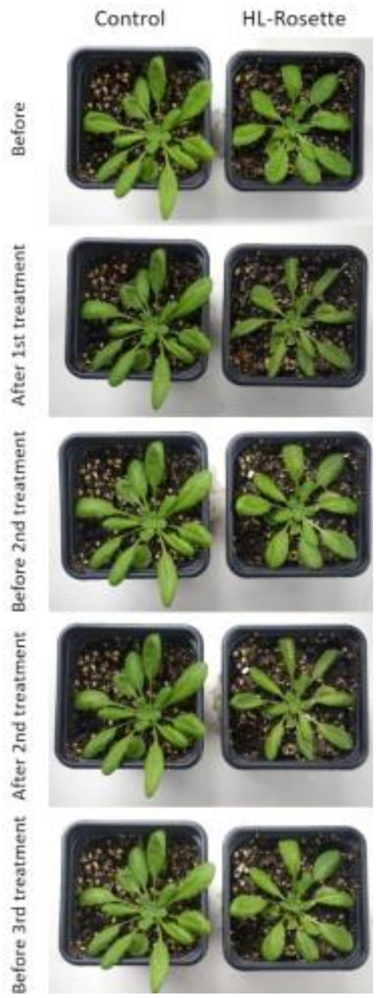

Figure S3. Visual responses of *Arabidopsis thaliana* plants on the 1st day treated by high light. The photos from top to bottom included the plant before treatment, after the 1st treatment for 30 min, before the 2nd treatment (recover from the 1st treatment for about 2.5 h), after the 2nd treatment and before the 3rd treatment (recover from the 2nd treatment for about 1.5 h).

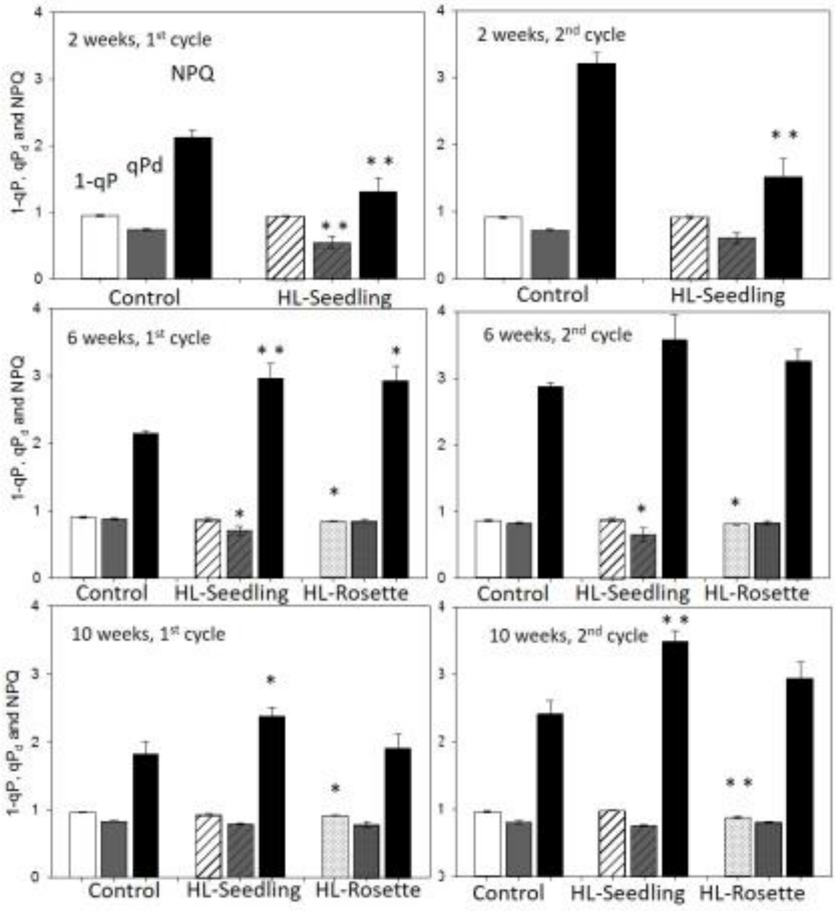

Figure S4. PAM chlorophyll fluorescence analysis of untreated (Control) and treated (HL-Seedling, coarse; HL-Rosette, dotted) *Arabidopsis thaliana* leaves after 2 weeks', 6 weeks' and 10 weeks' growth measured with actinic light 1150 µmol photons m^{-2} s^{-1} illumination. The excitation pressure (*1-qP*, white bar), photochemical quenching measured in the dark (qP_d, dark gray bar) and non-photochemical quenching (NPQ, black bar) after the first illumination cycle and the second illumination cycle. Error bars show the SEM (n=3).

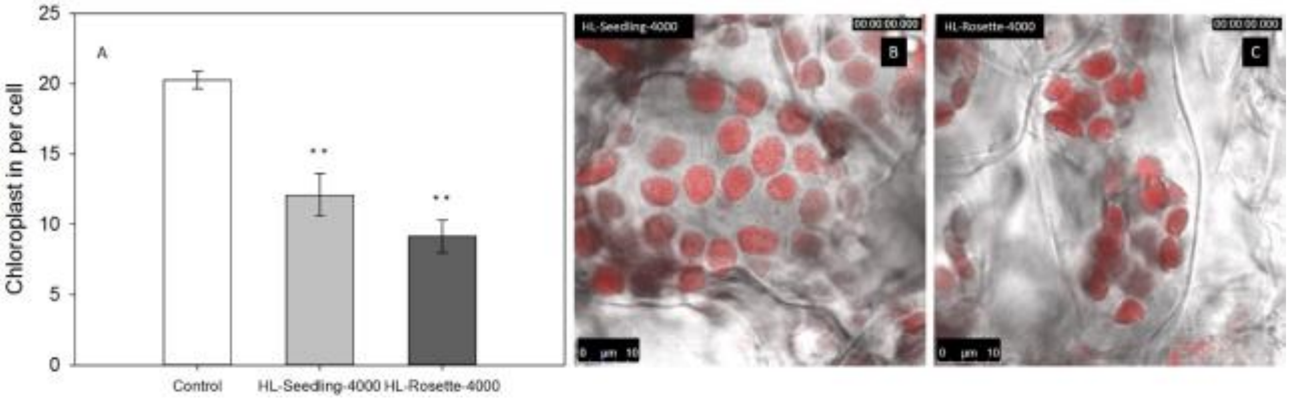

Figure S5. Chloroplast number and microscope of high light (4000 μmol photons $m^{-2}s^{-1}$) treated *Arabidopsis thaliana* leaves for 5 days from seedling (HL-Seedling-4000, light gray bar, plant age, 17 days) and rosette stage (HL-Rosette-4000, dark gray bar, plant age, 40 days). Untreated (control, white bar). (A) Chloroplast number. Error bars show the SEM (n=6-10). ** indicates the significant difference between control group and treated groups according to AVOVA ($p<0.01$). (B) Confocal microscope of chloroplasts in HL-Seedling-4000 plants, showing fluorescence of chlorophyll in red, scale bar 10 μm. (C) Confocal microscope of chloroplasts in HL-Rosette-4000 plants, showing fluorescence of chlorophyll in red, scale bar 10 μm.
